# Data on drying kinetics of a semi-automated gas-fired fish dryer

**DOI:** 10.1016/j.dib.2018.03.072

**Published:** 2018-03-21

**Authors:** Idehai O. Ohijeagbon, Olumuyiwa A. Lasode, Segun Adebayo, Oluseyi O. Ajayi, Olugbenga A. Omotosho

**Affiliations:** a Department of Mechanical Engineering, Covenant University, P.M.B 1023, Ota, Nigeria; b Department of Mechanical Engineering, University of Ilorin, P.M.B 1515, Ilorin, Nigeria

## Abstract

The dataset presented in this article represent the drying characteristics of a semi-automated gas-fired fish dryer. A cabinet dryer was constructed mainly from mild steel sheet metal and stainless steel, and was used for drying prepared fish samples of *Clarias gariepinus* species. Major operating parameters which included mass of fish, mass of Liquefied Petroleum Gas used, inside temperature of the dryer, and drying time were monitored. Also, output parameters such as the moisture content and energy utilization amongst others were observed and recorded at varying time intervals and specified drying temperatures. The drying temperature was sustained via an incorporated PID temperature controller that allowed drying to proceed until a relatively constant mass of the dried fish samples was attained. The information contained in this data article include a schematic drawing of drying kinetics analysis of the semi-automated gas-fired fish dryer and a pictorial view of the gas-fired cabinet dryer. Also included are pictorial representations of the washed and neatly folded degutted fish samples and dried fish samples. Data provided in this article are those relating to process parameters of the semi-automated fish dryer, data of output parameters of the fish dryer and comparisons of moisture content and energy utilization at different drying temperatures with time.

**Specifications Table**

**Table:** 

Subject area	*Mechanical Engineering*
More specific subject area	*Energy, thermodynamics, heat transfer, machine design*
Type of data	*Table, text, graph*
How data was acquired	*Data was obtained through measurements. A gas-fired cabinet dryer was developed to dry prepared fish samples at specified drying temperatures. The inside temperature of the dryer was measured with a type K thermocouple. The drying temperature was regulated with a PID temperature controller, model GL101G. The mass of Liquefied Petroleum Gas (LPG) used for firing was obtained by measuring the changes in mass of gas cylinder using a weighing scale, model SCALE BH-BS-1. A solenoid, model 121-070-0001 was used to control the inflow of LPG entering the burner and the changing mass of fish samples was obtained by measuring with a weighing scale: model HX-T*
Data format	*Raw, analyzed*
Experimental factors	*Freshly harvested set of fish samples were degutted, the fish samples were washed in saline water and then in clean water. The fish samples were weighed and their masses recorded. Each fish was bent into a circular shape and their heads clipped to their tails. Water was then allowed to drain for about ten minutes on a slab before the fish samples were finally ready to be placed in the dryer*
Experimental features	*The PID temperature controller was set to the desired working temperature after the LPG control knob was turned on and the burner in the dryer ignited. Upon getting to the set temperature, the solenoid valve opens thereby closing the passage of LPG into the burner, and causing a shutting down of the system except the pilot flame from the capillary tube. The pilot flame from the capillary tube ignites the burner whenever the thermocouple sends a signal to the PID temperature controller, indicating that the temperature in the dryer is lower than the set temperature. This process continues until the fish is completely dried without the need of any human intervention.*
Data source location	*Department of Mechanical Engineering, University of Ilorin, Ilorin, Nigeria*
Data accessibility	*Data are available within this article*

**Value of the data**

• The dataset is a potential source of information to conduct a performance assessment of fish drying systems.
• The data provided would be very useful to design fully automated fish dryers.
• The dataset could serve as a means of analyzing how to efficiently utilize energy effectively in fish drying operation and systems.
• The dataset could allow researchers gain insight into the thermo-economic benefits and potentials of fully automated fish dryers.

## 1 Data

**Fig. 1:**
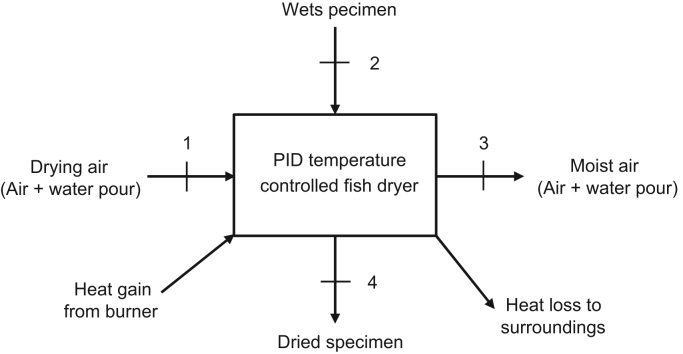
Schematic drawing of the analysis of drying kinetics of a semi-automated gas-fired fish dryer.

The data presented in this article includes process parameters, such as, mass of fish, mass of LPG used, inside temperature of the dryer and drying time [1]. The parameters listed in the data were collected at intervals of one hour drying time. The data enables the calculation of output parameters that include the moisture content [2] and energy utilization [3] at different specified drying temperatures of 80, 100 and 120 °C (Table 1, Table 2). The schematic drawing of the analysis of drying kinetics of the dryer is shown in Fig. 1.

**Table 1:** Data of process parameters of drying kinetics of a semi-automated gas-fired fish dryer.

Drying time (min)	Mass of fish (kg)			Mass of gas used (kg)			Temperature of the dryer (°C)		
	At various drying temperatures, *T_D_* (°C)								
	*T_D_* = 80	*T_D_* = 100	*T_D_* = 120	*T_D_* = 80	*T_D_* = 100	*T_D_* = 120	*T_D_* = 80	*T_D_* = 100	*T_D_* = 120
0	8.53	8.61	8.57	–	–	–	81.70	101.10	120.00
60	7.85	7.67	7.67	0.40	0.50	0.60	80.20	101.80	119.20
120	7.03	6.97	6.64	0.30	0.30	0.40	82.10	99.60	121.40
180	6.30	5.67	5.37	0.30	0.30	0.40	81.90	103.20	120.10
240	5.57	4.53	3.73	0.40	0.30	0.40	80.20	100.40	121.30
300	4.63	3.67	3.03	0.20	0.40	0.30	81.40	105.10	120.20
360	3.80	2.97	2.43	0.30	0.20	0.40	79.80	100.10	121.20
420	2.83	2.47	2.05	0.30	0.40	0.30	80.40	102.60	120.30
480	2.47	2.07		0.30	0.30		81.10	100.30	
540	2.03			0.40			80.10

**Table 2:** Data of output parameters of drying kinetics of a semi-automated gas-fired fish dryer.

Drying time (min)	Moisture content (kg water/kg dry fish)			Energy utilization (MJ)		
	At various drying temperatures, *T_D_* (°C)					
	*T_D_* = 80	*T_D_* = 100	*T_D_* = 120	*T_D_* = 80	*T_D_* = 100	*T_D_* = 120
0	0.770	0.750	0.798	2.310	3.483	5.697
60	0.678	0.670	0.667	3.047	4.812	7.726
120	0.586	0.580	0.571	3.930	7.077	9.241
180	0.483	0.489	0.429	5.421	8.977	11.044
240	0.414	0.364	0.226	7.968	10.077	12.850
300	0.299	0.239	0.167	9.831	12.244	16.592
360	0.218	0.159	0.095	11.888	15.077	17.816
420	0.092	0.068	0.000	13.943	16.985	21.070
480	0.046	0.000		15.190	19.977	
540	0.000			18.685		

## 2 Experimental design

**Fig. 2:**
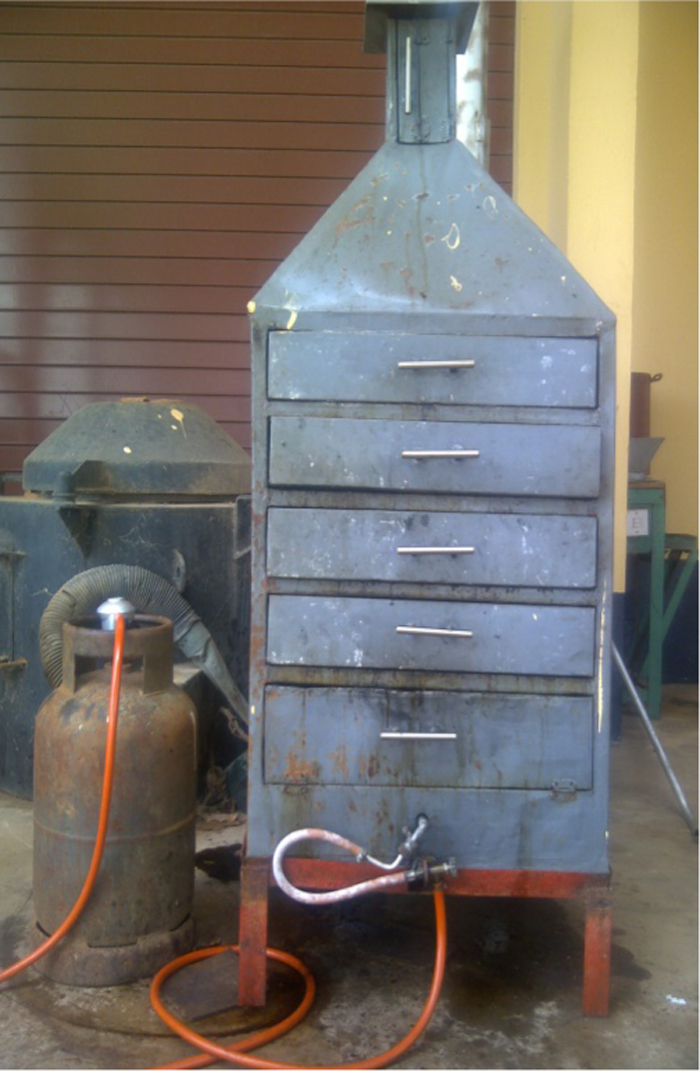
Pictorial view of the gas-fired cabinet dryer.

A developed gas-fired cabinet dryer made from 3.5 mm thick mild steel sheet metal was used for the experiment. The dimensions of the dryer are: height 140 cm, width 55 cm and breadth 60 cm. The dryer was mounted on a make-shift stand to elevate it from ground level as shown in Fig. 2. The height of the dryer comprises of three sections of major height 110 cm, conical section 20 cm and chimney 10 cm respectively. The major height was subdivided into five compartments, allowing for stainless steel wire-mesh trays or racks to accommodate the fish samples during drying operation [4] Fig. 2.

## 3 Materials and methods

**Fig. 3:**
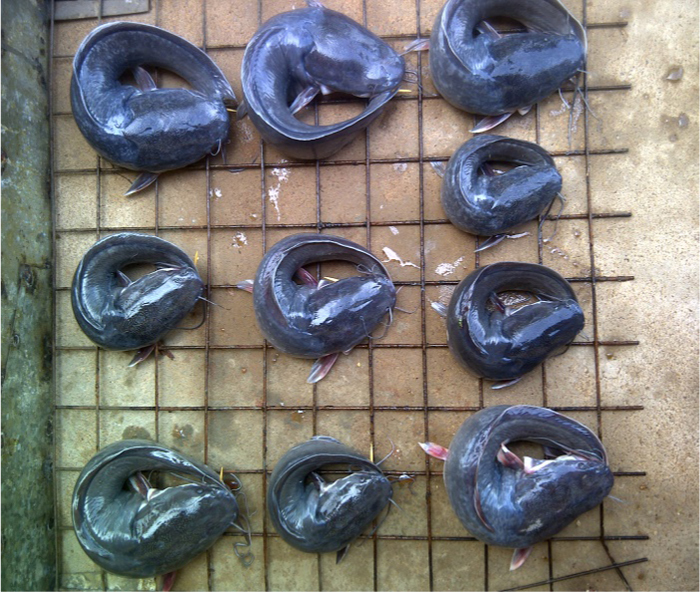
Degutted *Clarias gariepinus* fish samples, washed and neatly folded.

The dryer was pre-heated by opening the gas cylinder regulatory valve, igniting the burner and regulating until a blue flame was obtained. The dryer was preheated for 10 min to a temperature of 50 °C. Degutted *Clarias gariepinus* fish samples, washed and neatly folded were arranged on drying racks outside the dryer as shown in Fig. 3. The fish masses were weighed and recorded before loading into the dryer .

**Fig. 4:**
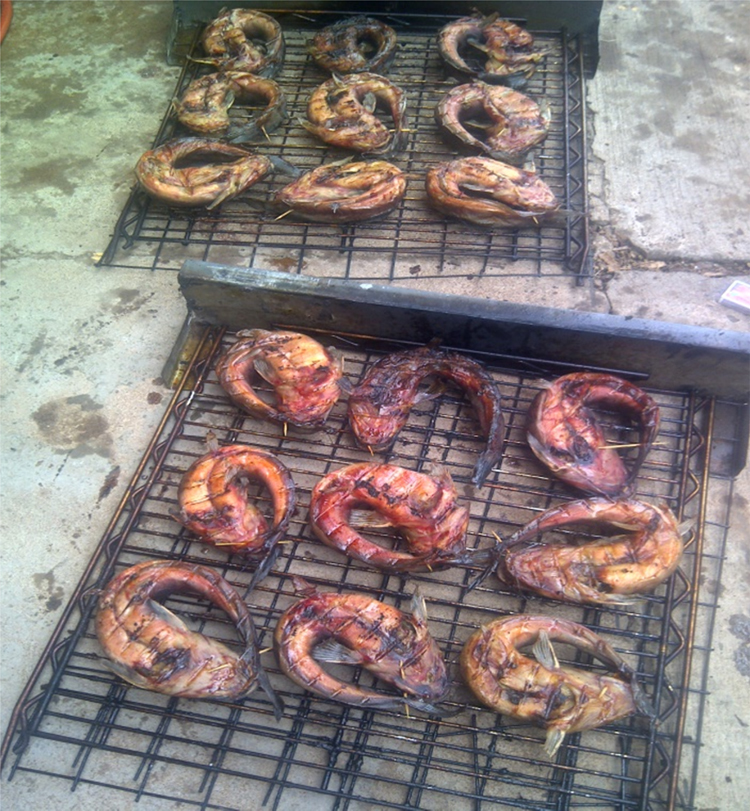
Dried *Clarias gariepinus* fish samples with belly faced up.

**Fig. 5:**
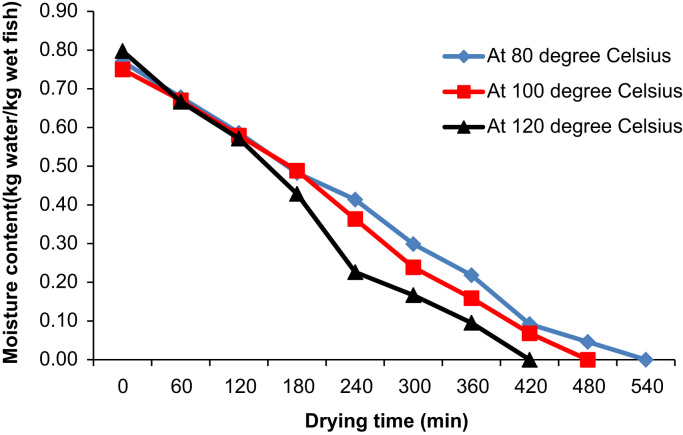
Comparison of moisture content with time at different drying temperatures.

**Fig. 6:**
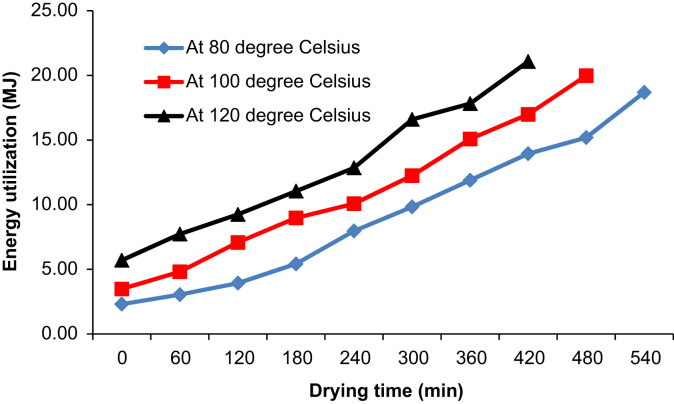
Comparison of energy utilization with time at different drying temperatures.

The experimental process was carried out by setting the PID temperature controller to 80 °C. The fish samples were then loaded into the dryer. Both the mass of the fish samples and LPG was measured after one hour. The mass of the fish samples and LPG was repeatedly measured every hour until the required dryness was attained. The compartments of the drying racks were interchanged after every 2 h and the fish samples turned over to allow for uniform drying. After the completion of drying of a set of fish samples, they were removed from the dryer and placed on a well-ventilated slab as shown in Fig. 4 in order to avoid moisture recapture due to condensation of escaping water vapor and heat. The experimental procedure was repeated by setting the PID temperature controller to 100 and 120 °C respectively. Data of operating parameters and ambient conditions of the dryer were captured and can be used to determine the exergetic efficiencies and irreversibility for enhanced dryer design, energy utilization and efficiency [5], [6], [7]. Comparisons of moisture content and energy utilization at different drying temperatures with time are shown in Fig. 5, Fig. 6. The moisture content on wet basis ($M_{w}$) is related to the initial mass ($m_{i}$) of wet samples at each time interval and the final dried mass ($m_{f}$) after the entire duration of drying. Consequently, the moisture content on wet basis was evaluated from Eq. (1):

(1) $$M_{w}=\frac{m_{i}-m_{f}}{m_{i}}$$
